# Transcriptomic Analysis and Functional Characterization Reveal the Duck Interferon Regulatory Factor 1 as an Important Restriction Factor in the Replication of Tembusu Virus

**DOI:** 10.3389/fmicb.2020.02069

**Published:** 2020-08-26

**Authors:** Chengwei Xiang, Mei Huang, Ting Xiong, Fang Rong, Linyu Li, Ding Xiang Liu, Rui Ai Chen

**Affiliations:** ^1^College of Veterinary Medicine, South China Agricultural University, Guangzhou, China; ^2^Zhaoqing Institute of Biotechnology Co., Ltd., Zhaoqing, China; ^3^Guangdong Province Key Laboratory of Microbial Signals and Disease Control, and Integrative Microbiology Research Centre, South China Agricultural University, Guangzhou, China; ^4^Zhaoqing Branch Center of Guangdong Laboratory for Lingnan Modern Agricultural Science and Technology, Zhaoqing, China

**Keywords:** Duck Tembusu virus, transcriptomic analysis, IRF1, VIPERIN, antiviral response

## Abstract

Duck Tembusu virus (DTMUV) infection has caused great economic losses to the poultry industry in China, since its first discovery in 2010. Understanding of host anti-DTMUV responses, especially the innate immunity against DTMUV infection, would be essential for the prevention and control of this viral disease. In this study, transcriptomic analysis of duck embryonic fibroblasts (DEFs) infected with DTMUV reveals that several innate immunity-related pathways, including Toll-like, NOD-like, and retinoic acid-inducible gene I (RIG-I)-like receptor signaling pathways, are activated. Further verification by RT-qPCR confirmed that RIG-I, MAD5, TLR3, TLR7, IFN-α, IFN-β, MX, PKR, MHCI, MHCII, IL-1β, IL-6, (IFN-regulatory factor 1) IRF1, VIPERIN, IFIT5, and CMPK2 were up-regulated in cells infected with DTMUV. Through overexpression and knockdown/out of gene expression, we demonstrated that both VIPERIN and IRF1 played an important role in the regulation of DTMUV replication. Overexpression of either one significantly reduced viral replication as characterized by reduced viral RNA copy numbers and the envelope protein expression. Consistently, down-regulation of either one resulted in the enhanced replication of DTMUV in the knockdown/out cells. We further proved that IRF1 can up-regulate VIPERIN gene expression during DTMUV infection, through induction of type 1 IFNs as well as directly binding to and activation of the VIPERIN promoter. This study provides a genome-wide differential gene expression profile in cells infected with DTMUV and reveals an important function for IRF1 as well as several other interferon-stimulated genes in restricting DTMUV replication.

## Introduction

Tembusu virus (TMUV) belongs to the genus Flavivirus, family Flaviviridae, Ntaya virus group. TMUV was firstly isolated in 1955 from Culex tritaeniorhynchus mosquitoes in Kuala Lumpur, Malaysia (Platt et al., 1975). In 2010, a novel duck disease characterized by acute egg-drop, depression, growth retardation and neurologic signs was emerged, and duck Tembusu virus (DTMUV) was confirmed to be the causative agent of the disease (Chen et al., 2014). DTMUV-infected ducks are frequently followed by a secondary bacterial infection, and the mortality can reach 5–20% (Su et al., 2011). Because of the zoonotic nature of Flaviviruses, DTMUV may also cause a public health concern. In fact, the virus can infect both avian and mammalian cells, and a number of people worked in the duck industry in China have shown seropositive to DTMUV (Tang et al., 2013a; Yu et al., 2018).

Duck Tembusu virus is a non-segmented and single-stranded RNA virus with the genome-length of 10990 nucleotides. The positive-sense RNA genome contains a single open frame (ORF) flanked by a type 1 capped 5′- non-coding region (NCR) and a 3′-NCR with no poly-A tail. This single ORF encodes one polyprotein precursor that is subsequently cleaved by viral and cellular proteases into three structural proteins, the capsid (C), precursor membrane (prM) and envelope (E), and seven non-structural proteins (NSs), NS1, NS2A, NS2B, NS3, NS4A, 2KNS4B, and NS5 (Tang et al., 2015; Zhu et al., 2015). In addition to their essential functions in the viral life cycles, both structural and non-structural proteins are probably involved in regulating the innate and adaptive immunity of host cells (Wu et al., 2019).

Host innate immunity is the first line of defense against viral infection. Once attached to the host cell, viral pathogen associated molecular patterns (PAMPs), including the protein and RNA components, are sensed by the host pattern recognition receptors (PRRs). PRRs mainly include retinoic acid-inducible gene I (RIG-I)-like receptors (RLRs), Toll-like receptors (TLRs), and nucleotide-binding oligomerization domain (NOD)-like receptors (NLRs). RLRs and TLRs mediate MAVS-dependent and TRIF/MyD88-dependent IFN signaling pathways, respectively. These signaling cascades recruit transcription regulators, such as TBK1 and IKKα/β, to activate NF-κB and IFN-regulatory factor (IRF) family members, resulting in the induction of type I IFNs, which in turn activate the expression of 1000 of IFN-stimulated genes (ISGs) (Gürtler and Bowie, 2013).

IFN-regulatory factor gene family encodes multiple transcription factors including IRF1, IRF3 and IRF7, and plays essential roles in host defense against viral infection through regulation of IFN expression (Tamura et al., 2008). So far, eleven IRF family members (IRF1-IRF11) have been identified in fish, nine (IRF1-IRF9) in mammals, and nine (IRF1-IRF11) in poultry with IRF3 and IRF9 missing (Stein et al., 2007). IRFs may regulate the expression of ISGs by directly binding to the specific IFN-stimulated regulatory elements (ISRE) (Mamane et al., 1999; Taniguchi et al., 2001). IRF1 can be induced by viral infection, dsRNA, IFNα/β or other cellular factors (Qian et al., 2018), and participates in antiviral and antibacterial responses, in hematopoietic differentiation and cytokine recognition (Nguyen et al., 1997; Li et al., 2015). Further studies demonstrated that IRF1 activates IFN expression against infection through the MyD88-dependent signaling pathway (Feng et al., 2015; Qian et al., 2018).

VIPERIN is an IFN-induced endoplasmic reticulum (ER)-associated antiviral protein and consists of three functional domains. Its N-terminal part contains an amphiphilic alpha helix, which mediates the localization of VIPERIN to the ER and lipid droplets; the central part contains the S-adenosylmethionine (SAM) domain of iron-sulfur [Fe/S] clusters; and the C-terminal domain may mediate the antiviral activity (Hinson and Cresswell, 2009; Duschene and Broderick, 2010; Shaveta et al., 2010; Helbig et al., 2011; Seo et al., 2011). Previous studies have shown that VIPERIN exhibits a broad-spectrum antiviral activity and can inhibit the replication of multiple members in the genus Flavivirus, such as tick-borne encephalitis virus (TBEV), West Nile virus (WNV), and dengue virus (DENV) (Jiang and Chen, 2011; Upadhyay et al., 2014). In vertebrates, the gene encoding cytidine monophosphate kinase 2 (CMPK2) is adjacent to the gene encoding VIPERIN. These two are transcribed together during IFN stimulation, indicating that they may have relevant antiviral functions (Minton, 2018).

IFN-induced Protein with Tetratricopeptide Repeats (IFIT) family is an ISG with antiviral functions. There are four IFIT members, IFIT1, IFIT2, IFIT3, and IFIT5, in the human genome, and three, IFIT1, IFIT2, and IFIT3, in rat. Whereas in birds, only one member, IFIT5, has been identified. Human IFIT5 may participate in the innate immunity, and in cells infected with infectious bursa disease virus (IBDV), IFIT5 was significantly up-regulated (Sun et al., 2015).

In this study, we used transcriptomic analysis to profile the differential expression of various cellular factors and the induction of signaling pathways in duck embryonic fibroblasts (DEFs) infected with DTMUV. We found that DTMUV infection induced drastic up-regulation of VIPERIN, IFIT5, CMPK2, and IRF1 expression at the mRNA level. Subsequent verification and characterization demonstrated that IRF1 can regulate the expression of VIPERIN directly, and IFIT5 and CMPK2 indirectly. Manipulation of the expression of these genes by knockout/knockdown and overexpression revealed that both duck IRF1 and VIPERIN, but not IFIT5 and CMPK2, could functionally restrict the replication of DTMUV. These results provide new insights into the activation of IFN signaling pathways and the important functions of some ISGs in cells infected with DTMUV.

## Materials and Methods

### Antibodies, Chemicals, and Reagents

Antibodies against β-actin (#4967), FLAG-tag (#2044), VIPERIN (#13996) were purchased from Cell Signaling Technology (Danvers, MA, United States), and anti-MYC was purchased from Transgen (Beijing, China). FITC (fluorescein isothiocyanate)-conjugated goat anti-mouse IgG and goat anti-rabbit IgG were purchased from Li-COR Biosciences (Lincoln, NE, United States). Monoclonal antibody against DTMUV E protein was prepared in the laboratory using the method described previously (Drew et al., 1995).

### Cells and Viruses

DF1 (chicken fibroblast cell line), BHK, Vero and 293T cells were cultured at 37°C with 5% CO_2_ in DMEM, supplemented with 10% fetal bovine serum (FBS) and 1% penicillin-streptomycin. DEF cells were prepared from 13-day old duck embryos as previously described (Shahsavandi et al., 2013).

Cells grown on 10 cm culture dishes were washed twice with serum-free medium and infected with DTMUV at an MOI of approximately 1 in serum-free medium. Mock controls were treated with the same amount of UV-inactivated DTMUV. After 2 h of absorption, cells were washed twice with serum-free medium and incubated with serum-free medium at 37°C till being harvested. Virus infection experiments were performed in a biosafety level 2 laboratory in the School of Veterinary Medicine, South China Agricultural University.

Using CRISPR/Cas9 technology, VIPERIN-knockout DF1 cell clone (DF1-viperin-KO cell) was generated by screening DF1 cells transfected with pX459-Viperin in the presence of 5 μg/ml puromycin. The knockout of VIPERIN in DF1-viperin-KO cell clone was confirmed by sequence verification, showing one extra adenosine (A) insertion between A_950_ and C_951_ (Supplementary Figure S2). The growth properties of DF1-viperin-KO cells were characterized, confirming that knockout of the gene did not result in detectable defects (Supplementary Figure S3).

Duck Tembusu virus strain QY17 (GenBank Accession No. MT447092) used in this study was previously isolated from an infected egg-laying duck in Qingyuan, Guangdong, China, and maintained in the laboratory. The strain was usually propagated in DEFs with a titer of 10^5.8^ TCID50/ml. Inactivation of DTMUV was performed by exposing the virus to 120,000 mJ/cm2 of 254-nm shortwave UV radiation for 15 min with a CL-1000 cross-linker (UVP). To demonstrate that the virus was indeed inactivated, DF1 cells were incubated with the UV-inactivated DTMUV (UV-DTMUV) and total lysates were analyzed by Western blot to confirm that no viral proteins can be detected.

### RNA Extraction From DEF Cells, Library Preparation, and Illumina HiSeq-X-Ten Sequencing

Duck embryonic fibroblasts were infected with DTMUV for 8, 16, and 24 h, respectively, and collected in triplicates, and the control cells treated with UV-DTMUV were collected in parallel. Total RNA was extracted by using TRIzol^®^ Reagent according to the manufacturer’s instructions (Invitrogen, Carlsbad, CA, United States), and the genomic DNA was removed by digestion with DNase I (TaKara, Kusatsu, Japan). The RNA quality was determined by using 2100 Bioanalyzer (Agilent, Santa Clara, CA, United States) and quantified with the ND-2000 (NanoDrop Technologies, Wilmington, DE, United States). Only RNA samples of high quality (OD260/280 = 1.8∼2.2, OD260/230 ≥ 2.0, RIN ≥ 6.5, 28S:18S ≥ 1.0, > 10 μg) were used to construct sequencing libraries.

RNA-seq transcriptome library was prepared with TruSeq™ RNA sample preparation Kit from Illumina (San Diego, CA, United States), followed the manufacturer’s instruction. 5 μg of each total RNA were used. The messenger RNA was isolated by oligo (dT) beads and then fragmented with fragmentation buffer. The double-stranded cDNA was synthesized by using a SuperScript double-stranded cDNA synthesis kit (Invitrogen, Carlsbad, CA, United States) with random hexamer primers (Illumina). The synthesized cDNA was then subjected to end-repair, phosphorylation and “A” base addition according to Illumina’s library construction protocol. Libraries were size-selected, and only the 200–300 bp cDNA fragments separated on 2% Low Range Ultra Agarose were amplified by using Phusion DNA polymerase (NEB, Ipswich, MA, United States) for 15 cycles. After quantified by TBS380, paired-end RNA-seq sequencing library was sequenced with the Illumina HiSeq-X-ten (2 × 150 bp read length).

### Read Mapping

The raw paired-end reads were trimmed and quality controlled by SeqPrep^1^ and Sickle^2^ with default parameters. The clean reads were separately aligned to reference genome with orientation mode of TopHat^3^ software (Trapnell et al., 2009). The mapping criteria of bowtie was as follows: sequencing reads should be uniquely matched to the genome allowing up to 2 mismatches, without insertions or deletions. Then the region of a gene was expanded following depths of sites and the operon was obtained. In addition, the whole genome was split into multiple 15 kbp windows that share 5 kbp. Newly transcribed regions were defined as more than two consecutive windows without overlapped region of the gene, where at least two reads mapped per window in the same orientation.

### Differential Expression Analysis and Functional Enrichment

To identify differentially expressed genes (DEGs) between DTMUV-infected and mock-treated DEF samples, the expression level of each transcript was calculated according to the fragments per kilo-base of exon per million mapped reads (FRKM) method. RSEM^4^ (Li and Dewey, 2011) was used to quantify gene abundance. R statistical package software Empirical Analysis of Digital Gene Expression in R (EdgeR)^5^ (Ti et al., 2015) was utilized for differential expression analysis. In addition, functional enrichment analyses including GO and KEGG were performed to identify which DEGs were significantly enriched in GO terms and metabolic pathways at Bonferroni-corrected *p*-value ≤ 0.05 compared with the whole-transcriptome background. GO functional enrichment and KEGG pathway analyses were carried out by Goatools^6^ (Xie et al., 2011).

### RNA Extraction and RT-qPCR Analysis

Total RNA was extracted by using the TRIzol reagent (Invitrogen, Carlsbad, CA, United States), and reverse-transcribed with the PrimeScript™ RT Master Mix (Takara, Kusatsu, Japan) according to the manufacturer’s instructions. The RT product (2 μl) was used for a quantitative PCR (qPCR) with the SYBR^®^ Premix Ex Taq™ II Kid according to the manufacturer’s instructions and the gene specific primers are listed in Supplementary data (Table S1). The qPCR analysis was performed using a QuantStudio 3 Real-Time PCR System (Applied Biosystems, Waltham, MA, United States). The standard protocol included enzyme activation at 50°C for 3 min, initial denaturation at 95°C for 3 min, followed by 40 cycles of denaturing (95°C, 5 s) and annealing/extension (60°C, 30 s) with fluorescent acquisition at the end of each cycle. The results were presented in the form of cycle threshold (CT) values. Using the 2^–ΔΔ*CT*^ method, the relative abundance of a transcript was calculated after normalizing to the internal GAPDH value. The gene specific primers for qPCR are listed in Supplementary Table S1.

### Plasmid Constructions

The expression plasmids, pXJ40-FLAG-VIPERIN. pXJ40-FLAG-CMPK2, pXJ40-FLAG-IFIT5, and pXJ40-MYC-IRF1, were constructed by inserting PCR products corresponding for each gene into a pXJ40-based plasmid. The nucleotide sequences of primers used to amplify these genes were listed in Supplementary Table S1.

Plasmid siCHECK2.0-VIPERIN which contains the LUC reportor gene driven by the VIPERIN promotor was purchased from Fenghui Biotechnology Co., Ltd. (Hunan, China).

Plasmid X459-Viperin used to knockout VIPERIN in DF1 cell was constructed by inserting the two complementary oligonucleotides (5′-*CACCg*GCAGCCTGATCAGGGAACGG-3′ and 5′-*AAAC*CCGTTCCCTGATCAGGCTGC*c*-3′), coding for the small guide RNA with the Bbs1 ends indicated in italic, into pX459. The small guide RNA was designed using an online program^7^.

### Transfection

Transfection of plasmid DNA was performed using TransIntro™ EL Transfection Reagent (Transgen, Beijing China) according to the manufacturer’s instructions. Briefly, cells were plated in a 12-well plate the day before transfection. For each transfection, 1.6 μg of plasmid DNA and 3 μl of TransIntro™ EL were diluted in 100 μl of plain medium, respectively, incubated for 5 min, and then mixed together by brief vortex. After incubation for another 15–20 min, the culture medium was changed with 800 μl of FBS-free DMEM and the transfection mixture was added. Cells were incubated at 37°C for 4–6 h before replacement of the transfection mixture with the complete medium. At 24 h post-transfection, cells were infected with DTMUV at an MOI of 1 or mock-treated with UV-DTMUV, and incubated till harvest at 8, 16, 24, 36, and 48 hpi, respectively, for protein and/or RNA extraction.

### Luciferase Reporter Assay

A 293T cells were plated in six well plates at 2.5 × 10^5^/2 ml and co-transfected with reporter plasmid psiCHECK2.0-VIPERIN (1.4 μg/well) and the control plasmid pXJ40-MYC (0.6 μg/well), or co-transfected with psiCHECK2.0-VIPERIN (1.4 μg/well) and pXJ40-MYC-IRF1 (0.6 μg/well) by using TransIntro™ EL kit. After 24 h transfection, cells were lysed and luciferase activity was measured by using a dual reporter luciferase assay kit (Promega, Madison, WI, United States), following the manufacturer’s instructions. The renilla luciferase activity was used for normalization. All reporter assays were repeated three times and the average value was presented.

### RNA Interference

IFN-regulatory factor 1 siRNA (+): 5′-GCACCAGUGAUCUGUACAATT-3′, siRNA (−): 5′-UUGUACAGAUCA CUGGUGCTT -3′ and NC siRNA (+): 5′-UUCUCCGAACGUGUCACGUTT-3′, siRNA (−): 5′-ACG UGACACGUUCGGAGAATT were purchased from Sangon Biotech (Shanghai, China). Transfection of siRNA was performed using TransIntro™ EL transfection reagent according to the manufacturer’s instructions. At 6 h post-transfection, cells were infected with DTMUV at an MOI of 1 or mock-treated, and continued to incubate till harvest at 8, 16, 24, 36, and 48 hpi, respectively.

### SDS-PAGE and Western Blot Analysis

Cells were harvested at 8, 16, 24, 36, and 48 hpi, respectively, by using cell scrapers (Corning, New York, NY, United States) and centrifuged at 15,000 × *g* for 1 min, the supernatant was discarded and the pellets were lysed in 1 × RIPA buffer. The cell lysates were centrifuged, the supernatants collected, and protein concentrations were measured. The cell lysate (25 μg protein) was then mixed with 5 × Laemmli sample buffer (0.3125 M Tris–HCl, pH 6.8, 10% SDS, 50% glycerol, 25% β-mercaptoethanol, and 0.025% bromophenol blue) and heated at 90°C for 5 min before separating on the sodium dodecyl sulfate-12% polyacrylamide gel by electrophoresis using the Bio-Rad Mini- PROT EAN Tetra cell system. The resolved proteins were then transferred onto a nitrocellulose membrane using the Bio-Rad TransBlot protein transfer system. The membrane was blocked with 5% BSA in TBST (20 mM Tris–HCl pH 7. 4, 150 mM NaCl, 0.1% Tween 20) for 1 h at room temperature, then incubated with 1 μg/ml primary antibodies at 4°C overnight. After washing with TBST, the membrane was incubated with 1:15000 diluted fluorescein isothiocyanate-conjugated goat anti-mouse IgG or goat anti-rabbit IgG at room temperature for 1 h. After washing with TBST, the proteins on the membrane were detected with the Azure c600 imager according to the manufacturer’s instructions. The proteins were quantified by densitometric measurement using NIH software Image J^8^. All experiments were repeated three times with similar results and one representative result is shown.

### Statistical Analysis

The one-way ANOVA method was used to analyze the significant difference between the indicated sample and the respective control sample. Significance levels were presented by the *p*-value (ns, non-significant; ^∗^*p* < 0.05; ^∗∗^*p* < 0.01; ^∗∗∗^*p* < 0.0001).

## Results

### Replication of DTMUV in Both Avian and Mammalian Cells

**FIGURE 1:**
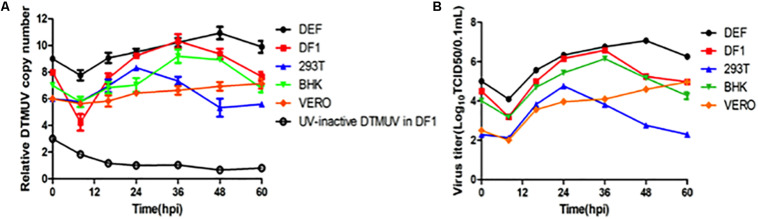
Replication of DTMUV in avian and mammalian cells. **(A)** Replication efficiencies of DTMUV in avian and mammalian cells as determined by the copy numbers of the viral genomic RNA by RT-qPCR. BHK-21, Vero, 293T, DF-1, and DEF cells were infected with DTMUV at an MOI of approximately 1, harvested at the indicated time points, and subjected to RNA extraction. Equal amounts of total RNA were reverse-transcribed. The levels of DTMUV genomic RNA were determined by RT-qPCR, and the average value was obtained from three repeats. **(B)** Replication efficiencies of DTMUV in avian and mammalian cells as determined by TCID50. BHK-21, Vero, 293T, DF-1, and DEF cells were infected with DTMUV at an MOI of approximately 1, harvested at the indicated time points, and the viral titers were determined as TCID50.

BHK-21, Vero, 293T, DF1, and DEF cells were infected, respectively, with DTMUV strain QY17 at an MOI of approximately 1, and DF1 cells treated with UV-DTMUV were included as a mock control. Total RNAs were extracted from cells harvested at the indicated time points, and viral gRNAs were detected by RT-qPCR. The results showed that DTMUV QY17 was able to infect all of these cell types with different efficiencies (Figure 1). DTMUV QY17 replicated with higher efficiencies in DEF, DF1, and BHK-21 cells than did in Vero and 293-T cells (Figure 1A). Viral replication in DEFs was faster than that in other cell types with significantly higher viral RNA copy numbers at each time point, reaching more than 10^10^ copy numbers at 48 hpi. The virus grew slowly in Vero cells and could not reach the peak even at 72 hpi (Figure 1A). In 293T cells, although the viral replication reached the peak earlier, the peak copy numbers were obviously lower than those in other three cell types (Figure 1A). Virus titers in cell culture supernatants were determined by TCID50 assay, showing consistent data with the RNA copy numbers as determined by RT-qPCR (Figure 1B). These results confirm that DTMUV QY17 can replicate in multiple mammalian and avian cells, and DEFs are the most permissive cell type for DTMUV replication.

### Transcriptomic Analysis of Differential Gene Expression in DEFs Infected With DTMUV

**FIGURE 2:**
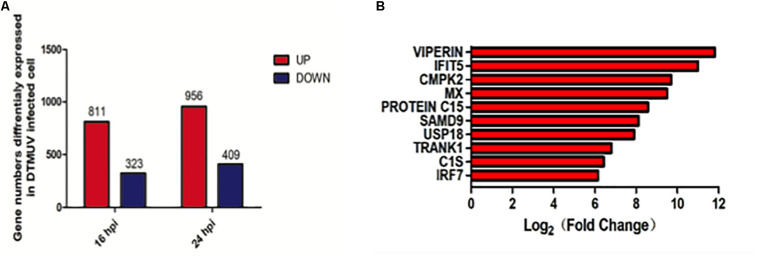
Transcriptomic analysis of differential gene expression in DEF cells infected with DTMUV. **(A)** The up-regulated gene numbers in DTMUV-infected cells at 16 or 24 hpi were shown in red, and those down-regulated in blue. **(B)** The top 10 genes with highest expression levels among all the up-regulated genes from the total transcriptomic data.

Duck embryonic fibroblasts were infected with DTMUV QY17 and harvested at 16 and 24 hpi, respectively. Total RNAs were extracted and subjected to transcriptomic analysis. DEFs treated with UV-DTMUV were included as a mock control. The expression of host genes from the infected and mock-treated cells was normalized to the internal GAPDH transcript, and the ratio of each transcript at each time point was calculated and presented under the threshold of *p*-value < 0.05 and |log2 (fold change)| > 1. Among a total of 1134 differentially regulated genes at 16 hpi, 811 were up-regulated and 323 down-regulated; and among 1365 differentially regulated genes at 24 hpi, 956 were up-regulated and 409 down-regulated (Figure 2A). Among all the up-regulated genes from the total transcriptomic data, the 10 genes with highest expression levels are VIPERIN, IFIT5, CMPK2, MX, PROTEIN C15, SAMD9, USP18, TRANK1, C1S, and IRF3 (Figure 2B).

### Biologic Significance of Differentially Expressed Genes in DEFs Infected With DTMUV

**FIGURE 3:**
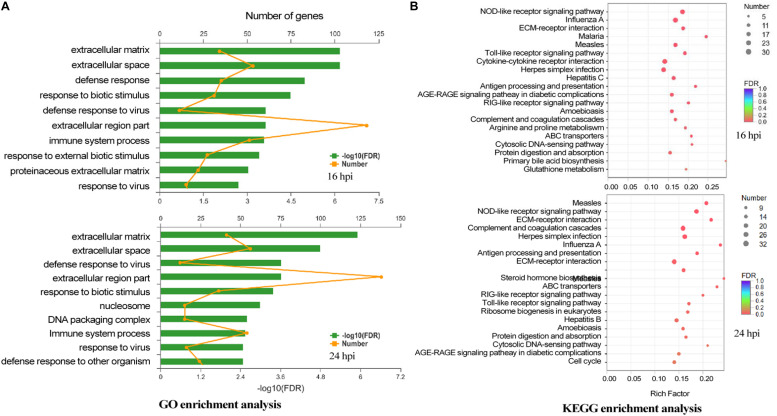
The biologic significance of differentially expressed genes in DEF cells infected with DTMUV. **(A)** Genes induced by DTMUV infection at 16 hpi (upper panel) and 24 hpi (lower panel), respectively, were classified with the GO system. **(B)** Genes induced by DTMUV infection at 16 hpi (upper panel) and 24 hpi (lower panel), respectively, were classified with the KEGG system.

The biologic significances of these genes in DTMUV infection were then analyzed. Through Gene Ontology (GO) system, these genes were classified, showing that genes regulated by DTMUV infection were mostly clustered at extracellular matrix, extracellular space and nucleosome, and functionally involved in defense response to viral infection, biotic stimulus, DNA packaging and immune response (Figure 3A).

By Kyoto Encyclopedia of Genes and Genomes (KEGG) analysis, these genes were shown to be enriched in systemic lupus erythematosus, NLR signaling pathway, complement and coagulation cascades, herpes simplex infection and influenza A antigen processing (Figure 3B).

We further analyzed these genes in five representative immune-relevant pathways including TLRs, RLRs, NLRs, chemokine, and hepatitis signaling pathways. The top 10 most up-regulated genes in each pathway were listed in Table 1 and in the Supplementary data (Figures S1A–E). These include: IRF7, IFNA, IFNB, STAT1, TLR3, PI3K3CD, FADD, NFKBIA, IKKE, and IFNAR2 in the TLR signaling pathway; IRF7, IFNB, IFNA, RIG-I, MDA5, MITA, TREM25, LGP2, FADD, and NFKBIA in the RLR signaling pathway; IRF7, GBP-1, IFNB, IFNA, GBP-2, STAT1, MITA, RIPK2, TRAF5, and P2RX7 in the NLR signaling pathway; STAT1, CX3CL, ADCY8, CCL19, PI3K3C, RAC2, FAK2, DOCK2, IL-8, and PRKCB in the chemokine signaling pathway; and IRF7, IFNB, IFNA, RIG-I, IRF1, MDA5, STAT1, TLR3, PIK3CA, and NFKBIA in the hepatitis pathway (Table 1). As presented in Figure 2B, the top 10 up-regulated genes are VIPERIN, IFIT5, CMPK2, MX, PROTEIN C15, SAMD9, USP18, TRANK1, C1S, and IRF3 (see Figure 2B).

**TABLE 1:** Top 10 up-regulated genes in each of the Toll-like receptors (TLR), RLG-L-like receptors (RLR), Nod-like receptors (NLR), chemokine, and hepatitis signaling pathways activated by DTMUV replication in DEF cells.

**Order**	**TLR**		**RLR**		**NLR**		**Chemokine**		**Hepatitis**	
	**16 h**	**24 h**	**16 h**	**24 h**	**16 h**	**24 h**	**16 h**	**24 h**	**16 h**	**24 h**
1	IRF7	IRF7	IRF7	IRF7	IRF7	IRF7	CX3CL	STAT1	IRF7	IRF7
2	STAT1	IFNB	TRIM25	IFNB	GBP1	GBP1	STAT1	CX3CL	IRF1	IFNB
3	IL8	IFNA	MITA	IFNA	MITA	IFNB	CCL20	ADCY8	STAT1	IFNA
4	IFNA	STAT1	RIG-I	RIG-I	STAT1	IFNA	ADCY8	CCL19	RIG-I	RIG-I
5	CCL5	TLR3	LGP2	MDA5	RIPK2	GBP2	RAG2	PIK3CA	MDA5	IRF1
6	NFK6IA	PIK3CA	MDA5	MITA	GBP2	STAT1	IL8	RAC2	IL8	MDA5
7	TLR3	FADD	IL8	TRIM25	TRPV2	MITA	CCL5	NFKBIA	IFNA	STAT1
8	IFNB	NFKBIA	IFNA	LGP2	TRAF5	RIPK2	NFKBIA	PTK2B	NFKBIA	TLR3
9	PIK3C	IKBKE	NFKBI	FADD	IL8	TRAF5	CCL19	DOCK2	TLR3	PIK3CA
10	IKBKE	IFNAR2	IFNB	NFKBIA	TNFAIP	TRPV2	PIK3CA	IL8	IFNB	FADD

The innate immunity-related genes induced by DTMUV infection were then checked by RT-qPCR to validate the expression profiles of the up-regulated genes based on the transcriptomic data.

### Verification of the Transcriptomic Data by RT-qPCR

**FIGURE 4:**
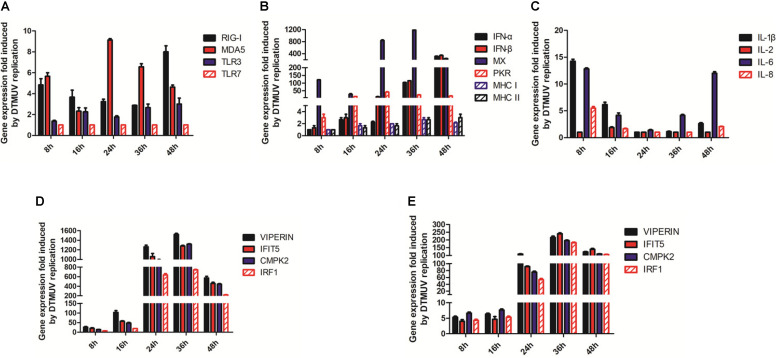
RT-qPCR verification of the up-regulated genes involved in the innate immunity by DTMUV infection. **(A)** Fold-inductions of RIG-1, MAD5, TLR3, and TLR7 in DEFs by DTMUV infection. **(B)** Fold-inductions of IFN-α, IFN-β, MX, PKR, MHCI, and MHCII in DEFs by DTMUV infection. **(C)** Fold-inductions of IL-1β, IL-2, IL-6, and IL-8 in DEFs by DTMUV infection. **(D)** Fold-inductions of VIPERIN, IFIT5, CMPK2, and IRF1 in DEFs by DTMUV infection. **(E)** Fold-inductions of VIPERIN, IFIT5, CMPK2, and IRF1 in DF1 cells by DTMUV infection.

Duck embryonic fibroblasts were infected with DTMUV and mock-treated with UV-DTMUV, respectively. Cells were harvested at the indicated time points, followed by RNA extraction and RT-qPCR analysis. After normalization to the GAPDH transcript, the up-regulation of each gene in DTMUV-infected cells compared to that in mock-treated cells was calculated and shown as fold of induction (Figures 4A–D). Four key genes coding for PRRs in the IFN pathway, RIG-1, MDA5, TLR3, and TLR7 were 1–9 fold up-regulated in the infected cells (Figure 4A). IFN-α, IFN-β, MX, and PKR were greatly induced by DTMUV infection, with about 40- to more than 1000-fold induction; among them, the most prominent two were IFN-α and IFN-β (Figure 4B). MHCI and MHCII induction was relatively lower, at only 1- to 3-fold (Figure 4B). Up-regulation of interleukin genes, including IL-1β, IL-6, and IL-8, was also obvious; both IL-1β and IL-6 were more than 13-fold up-regulated at 8 hpi (Figure 4C). In addition, a second-wave induction of IL-6 was observed at 48 hpi (Figure 4C). IL-2 was marginally increased and IL-8 was about 6-fold increased at 8 hpi (Figure 4C).

Verification of VIPERIN, IFIT5, CMPK2, and IRF1 expression by RT-qPCR was also carried out, confirming that DTMUV infection highly induced the expression of these genes at the mRNA level. The transcript levels of these four genes were increased gradually and reached the peaks at 24–36 hpi in DEFs. As shown in Figure 4D, a 600- to 1200-fold up-regulation of the transcription of these genes was observed at 24 hpi, and a more than 1500-fold increase was observed when their up-regulation reached the peak at 36 hpi. These results validate the transcriptomic data.

Induction of VIPERIN, IFIT5, CMPK2, and IRF1 expression in DTMUV-infected DF1 cells by RT-qPCR was then carried out to confirm if DTMUV infection would also induce the expression of these genes in this cell type. As shown in Figure 4E, a similar, although at lower levels, induction kinetics for these genes was observed in DTMUV-infected DF1 cells, confirming the induction of these gene expression by DTMUV infection is not cell-type specific.

### Effects of Overexpression of VIPERIN, IRF1, IFIT5, and CMPK2 on DTMUV Replication in DF1 Cells

**FIGURE 5:**
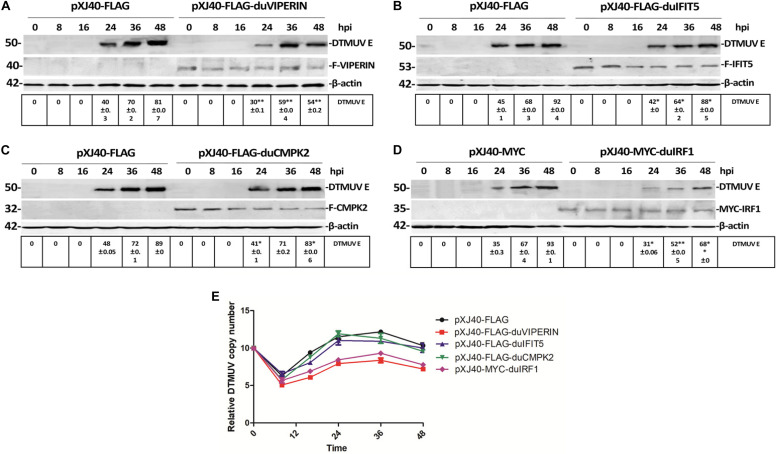
Effect of overexpression of VIPERIN, IRF1, IFIT5, and CMPK2 on DTMUV replication in DF-1 cells. **(A)** The viral E protein expressed in DTMUV-infected DF-1 cells transiently overexpressing duck VIPERIN. DF1 cells transiently expressing the FLAG-tagged VIPERIN were infected with DTMUV and harvested at indicated time points. Total lysates were prepared and proteins were separated by SDS-PAGE and detected by Western blot with anti-FLAG (VIPERIN) and anti-DTMUV E protein antisera, respectively. β-actin was included as a loading control. ^∗^*p* < 0. 05; ^∗∗^*p* < 0. 01. **(B)** The viral E protein expressed in DTMUV-infected DF-1 cells transiently overexpressing duck IFIT5. DF1 cells transiently expressing the FLAG-tagged IFIT5 were infected with DTMUV and harvested at indicated time points. Total lysates were prepared and proteins were separated by SDS-PAGE and detected by Western blot with anti-FLAG (IFIT5) and anti-DTMUV E protein antisera, respectively. β-actin was included as a loading control. ^∗^*p* < 0. 05; ^∗∗^*p* < 0. 01. **(C)** The viral E protein expressed in DTMUV-infected DF-1 cells transiently overexpressing duck CMPK2. DF1 cells transiently expressing the FLAG-tagged CMPK2 were infected with DTMUV and harvested at indicated time points. Total lysates were prepared and proteins were separated by SDS-PAGE and detected by Western blot with anti-FLAG (CMPK2) and anti-DTMUV E protein antisera, respectively. β-actin was included as a loading control. ^∗^*p* < 0. 05; ^∗∗^*p* < 0. 01. **(D)** The viral E protein expressed in DTMUV-infected DF-1 cells transiently overexpressing duck IRF1. DF1 cells transiently expressing the MYC-tagged IRF1 were infected with DTMUV and harvested at indicated time points. Total lysates were prepared and proteins were separated by SDS-PAGE and detected by Western blot with anti-MYC (IRF1) and anti-DTMUV E protein antisera, respectively. β-actin was included as a loading control. ^∗^*p* < 0. 05; ^∗∗^*p* < 0. 01. **(E)** Shown are the genomic RNA levels of DTMUV in DF-1 cells overexpressing VIPERIN, IRF1, IFIT5, and CMPK2, respectively, at the indicated time points post-DTMUV infection. Error bars represent the standard errors from three repeated experiments in each time point.

The drastic up-regulation of VIPERIN, IFIT5, CMPK2, and IRF1 expression in DTMUV-infected cells prompted us to carry out the subsequent studies to address their functional significance in DTMUV replication as well as the underlying mechanisms. As it was difficult to transfect the primary DEFs, DF1 cells were used in the overexpression studies. DF1 cells transiently expressing the FLAG-tagged VIPERIN, CMPK2 and IFIT5 and the MYC-tagged IRF1, respectively, were infected with DTMUV, and harvested at indicated time points. Overexpression of VIPERIN, IFIT5, CMPK2, and IRF1 was confirmed by Western blot with anti-FLAG or anti-MYC antibodies, and the expression of DTMUV E protein was detected by Western blot with antiserum against this protein, indicative of the level of viral replication. Overexpression of duck VIPERIN and IRF1, respectively, reduced the E protein level at 24 and 36 hpi, but overexpression of IFIT5 and CMPK2 showed only minor effect on DTMUV replication (Figures 5A–D). RT-qPCR analysis of the mRNA level of DTMUV E protein confirmed a 10^2^–10^2.5^-fold reduction of viral RNA levels in cells overexpressing VIPERIN and IRF1 (Figure 5E). Overexpression of duck IFIT5 and CMPK2 did not significantly affect viral RNA levels (Figure 5E). These results suggest that both VIPERIN and IRF1 may play a role in restriction of DTMUV replication in DF1 cells.

### Effects of Knockdown/Out of VIPERIN and IRF1 on DTMUV Replication in Avian and Mammalian Cells

**FIGURE 6:**
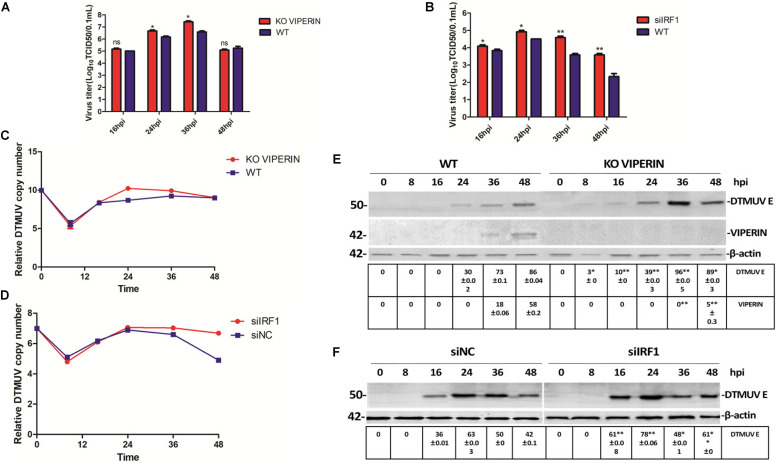
Effect of knockdown/out of VIPERIN and IRF1 on DTMUV replication in DF-1 cells. **(A)** Viral titers from DTMUV-infected wild type (blue, WT) and VIPERIN-knockout DF-1 cells (red, KO VIPERIN) harvested at the indicated times, respectively. *T* test between the red and blue groups was done. ^∗^*p* < 0. 05; ^∗∗^*p* < 0. 01; ns, non-significance. **(B)** Viral titers obtained from 293T cells transfected with siNC (blue, WT) or siIRF1 (red, siIRF1), followed by DTMUV infection, and harvested at the indicated times. *T* test between the red and blue groups was done. ^∗^*p* < 0. 05; ^∗∗^*p* < 0. 01; ns, non-significance. **(C)** Relative viral RNA levels from VIPERIN-knockout (KO VIPERIN) or wild type DF-1 cells (WT) harvested at the indicated times post-DTMUV infection. **(D)** Relative viral RNA levels from IRF1-knockdown (siIRF1) or the negative control (siNC) 293T cells harvested at the indicated times post-DTMUV infection. **(E)** The E protein expressed in WT or KO VIPERIN DF-1 cells at the indicated times post-DTMUV infection. The values of E protein and VIPERIN were presented after normalized to β-actin. ^∗^*p* < 0. 05; ^∗∗^*p* < 0. 01. **(F)** The E protein expressed in 293-T or IRF1-knockdown 293T cells at the indicated times post-DTMUV infection. The value of E protein was presented after normalized to β-actin. ^∗^*p* < 0. 05; ^∗∗^*p* < 0. 01.

As overexpression of both VIPERIN and IRF1 appears to significantly suppress the replication of DTMUV, transient knockdown/out of IRF1 in 293T cells with siRNA was carried out. Meanwhile, successful knockout of VIPERIN in DF1 cells by CRISPR-cas9 was achieved and a stable knockout cell clone (DF1-viperin-KO) was obtained. After the IRF1-knockdown 293T cells and DF1-viperin-KO were infected with DTMUV, culture media and total cells were collected at indicated time pints post-infection. The viral particles released to the culture media were titrated with TCID50 assay and the levels of viral RNA in the infected cells were determined by RT-qPCR. As shown in Figure 6A, virus titers in the culture media collected from the knockdown/out cells were obviously higher than those from the negative control cells. Virus titers from DF1-viperin-KO were 5- to 10-fold higher at 24 and 36 hpi, respectively, than those from wild type cells (Figure 6A). Virus titers from IRF1-knockdown cells were 10- to 30-fold higher at 36 and 48 hpi, respectively, than those from the negative control cells (Figure 6B). RT-qPCR results shown in Figures 6C,D confirmed 10- to 100-fold increases of viral RNA levels in DF1-viperin-KO cells at 24 and 36 hpi, and 5- to 100-fold increases in IRF1-knockdown cells at 24 and 48 hpi, respectively. These results were further confirmed by Western blot shown in Figures 6E,F. As anti-duIRF1 antibodies were not available, RT-qPCR was conducted instead, confirming that IRF1 was efficiently silenced (data not shown). These results further demonstrate the involvement of VIPERIN and IRF1 in regulation of DTMUV replication in culture cells.

### DTMUV Infection-Induced Up-regulation of VIPERIN, IFIT5, and CMPK2 Is Regulated by IRF1

**FIGURE 7:**
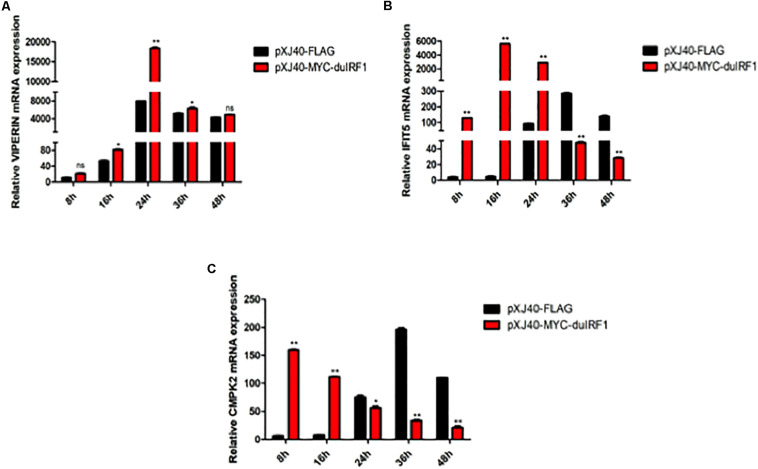
Regulation of DTMUV infection-induced up-regulation of VIPERIN, IFIT5, and CMPK2 by overexpression of duck IRF1. The relative mRNA levels of VIPERIN **(A)**, IFIT5 **(B)**, and CMPK2 **(C)** in DF-1 cells overexpressing duck IRF1. DF-1 cells were transfected with pXJ40-MYC-IRF1 and pXJ40-FLAG for 16 h, respectively, and infected with DTMUV at an MOI of approximately 1. Cells were harvested at the indicated time points for RT-qPCR with gene specific primers for each gene to determine the relative mRNA levels. *p < 0.05, **p < 0.01.

As a known transcriptional activator or repressor of a diversity of target genes, IRF1 may regulate the induction of VIPERIN, IFIT5, and CMPK2 in DTMUV-infected cells. This possibility was firstly studied by checking the effect of IRF1 overexpression on the induction of VIPERIN, IFIT5, and CMPK2 in DF1 cells infected with DTMUV. As shown in Figure 7A, overexpression of IRF1 resulted in a 1.8 × 10^4^ -fold higher peak level of VIPERIN. Overexpression of IRF1 induced much earlier expression of IFIT5, reaching the peak at 16 hpi and with a 5 × 10^3^ -fold higher level than that in the control cells (Figure 7B). CMPK2 induction was also much earlier in cells overexpressing IRF1, with the peak expression level at 8 hpi, which was 28 h earlier than that in the control cells (Figure 7C).

**FIGURE 8:**
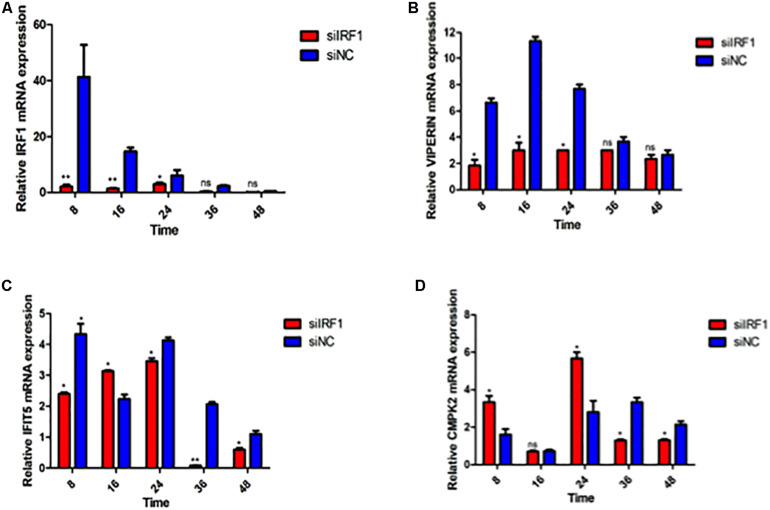
Regulation of DTMUV infection-induced up-regulation of VIPERIN, IFIT5, and CMPK2 by knockdown of IRF1. The relative mRNA levels of IRF1 **(A)**, VIPERIN **(B)**, IFIT5 **(C)**, and CMPK2 **(D)** in IRF1-knockdown 293T cells infected with DTMUV. 293T cells were transfected with siIRF1 and siNC for 6 h, respectively, and infected with DTMUV at an MOI of approximately 1. Cells were harvested at the indicated time points for RT-qPCR with gene specific primers for each of the four genes to determine the relative mRNA levels. ^∗^*p* < 0. 05; ^∗∗^*p* < 0. 01; ns, non-significance.

Knockdown of IRF1 by siRNA in 293T cells was then carried out. The knockdown efficiency and the expression levels of VIPERIN, IFIT5, and CMPK2 in the infected cells were examined by RT-qPCR. As shown in Figure 8A, efficient knockdown of the IRF1 expression was achieved. DTMUV infection-induced expression of VIPERIN was much reduced in IRF1-knockdown cells, compared with that in siNC-transfected cells (Figure 8B), but IRF1-knockdown only showed a very mild effect on IFIT5 expression (Figure 8C). The induction of CMPK2 in IRF1-knockdown cells infected with DTMUV displayed two peaks with the first one at 8 hpi and the second one at 24 hpi (Figure 8D). The relative values of both peaks were 2-fold lower than those in the control, but with only slightly lower levels of induction in other time points (Figure 8D). Taken together, these results demonstrate that IRF1 may regulate the induction of VIPERIN in DTMUV-infected cells.

### IRF1 Directly Activates the VIPERIN Promoter

**FIGURE 9:**
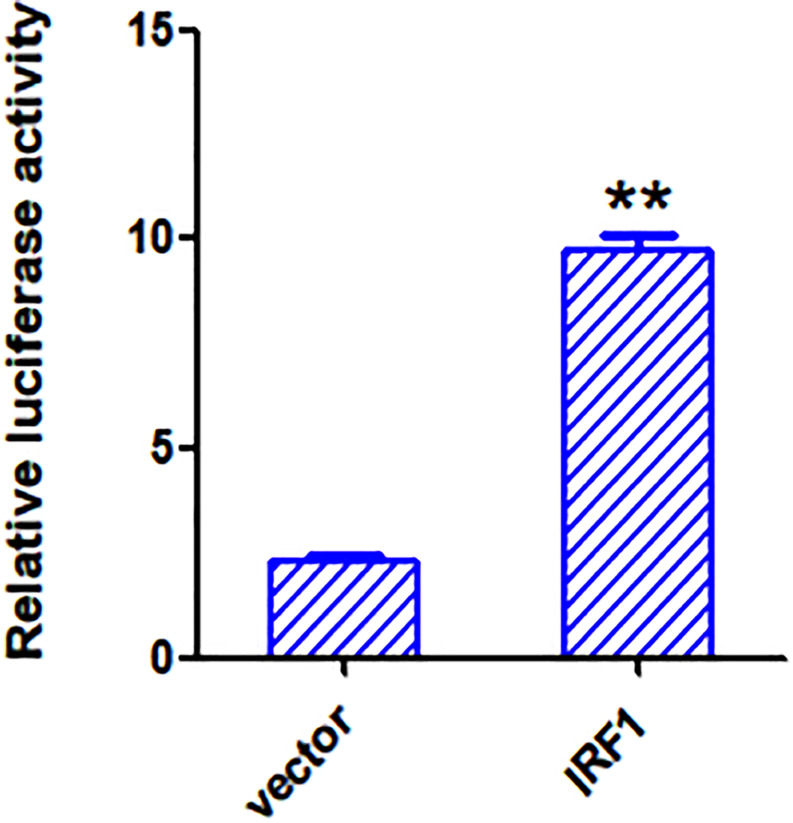
Overexpression of IRF1 activates the VIPERIN promoter. The luciferase activities produced in psiCHECK2.0-VIPERIN-transfected 293T cells overexpressing IRF1 or an empty vector. **p < 0. 01.

To see if IRF1 acts as a direct transcription activator on the VIPERIN gene promoter, a luciferase reporter system, in which the luciferase expression is directly driven by the VIPERIN gene promoter, was generated. Dual reporter luciferase assay was conducted in cells with or without overexpression of IRF1. As shown in Figure 9, the luciferase activity was 3-fold higher in cells overexpressing IRF1 compared with cells overexpressing the vector control. This result confirms that IRF1 may enhance the VIPERIN gene promoter-driven reporter gene expression, possibly by directly binding to the promoter region.

## Discussion

The innate immune system is the first line of host defense against infection by pathogenic microorganisms, including DTMUV (Li et al., 2020). Previous studies have shown that DTMUV is mainly recognized by TLR3 and MDA5, resulting in the induction of IFNs and various other antiviral proteins (Yu et al., 2018). In this study, we demonstrate that internalization and replication of DTMUV in DEFs lead to the activation of several innate immune-related signaling pathways. VIPERIN, CMPK2, and IFIT5 were found to be the most up-regulated genes, consistent with a previous study by Hu et al. (2019). Further functional characterization reveals that IRF1 may play an important role in restriction of DTMUV replication. In addition to its role in the induction of type I IFNs, IRF1 may induce the expression of VIPERIN by directly binding to the VIPERIN promoter region. This contributes to the up-regulation of VIPERIN in DTMUV-infected cells. Some other viruses, such as human cytomegalovirus (HCMV) and Vesicular Stomatitis Virus (VSV), were also found to directly induce the expression of VIPERIN by IRF1 or IRF3, independently of the IFN pathway (Stirnweiss et al., 2010; Seo et al., 2011). This is different from the classical ISG induction pathway, when VIPERIN expression was induced by Poly I: C, LPS, dsDNA and several viruses, including Sendai virus, Sindbis virus and Pseudorabies virus (Bowie and Unterholzner, 2008).

As a member of IRF transcription factor family that regulates IFN expression during viral infection, IRF1 plays a very important role in regulating the expression of IFNs and ISGs, in addition to NF-κB, IRF3, and IRF7 (Tamura et al., 2008; Feng et al., 2015). The whole family of IRF proteins contains a conserved N-terminal DNA binding domain (DBD) of approximately 115 amino acids, involving five or six conserved tryptophan (Trp) residues that form a helix-loop-helix motif (Escalante et al., 1998), and a less-well-conserved C-terminal region with specific functions for each IRF (Mamane et al., 1999). IRF1 expression in cells can be induced by virus infection and various cytokines as well as other stimuli, such as dsRNA, IFN-α/β, TNF, and IL-1, in different cell types. Its N-terminal DBD recognizes similar DNA sequences, including ISRE and IFN regulatory elements (IRF-E). Previous studies have demonstrated that duck IRF1 and IRF7 can activate the expression of duck IFN-β in a complementary manner through MyD88-dependent signaling pathway, and overexpression of duck IRF1 leads to the up-regulation of a number of duck ISGs, including IFITM1, OASL, and 1SG12-2 (Qian et al., 2018). As a broad antiviral transcription factor, IRF1 can trigger the expression of IFNs and ISGs, effectively inhibiting the replication of a variety of RNA and DNA viruses (Schoggins et al., 2011). Overexpression of duck IRF1 can effectively inhibit the replication of four avian viruses in DEFs, including H5N1, H9N2, NDV, and DTMUV (Qian et al., 2018). IRF1 can also directly bind to the promoter region of STAT1 and induce STAT1 transcription and phosphorylation, leading to the activation of the transcription of antiviral ISGs (Xu et al., 2016). Data presented in this study demonstrate that duck IRF1 may directly bind to and activate the duck VIPERIN promoter, suggesting the presence of conserved IRF1-binding motifs in this promoter region. In fact, examination of the duck VIPERIN promoter sequence reveals the presence of the 18-nucleotide core IRF1 binding motif (RAAASNGAAAGTGAAASY) (Shi et al., 2011). Further characterization of the binding specificity and kinetics would provide mechanistic details underlying the activation of duck VIPERIN by IRF1 and their antiviral functions during DTMUV infection.

In addition to IRF1, Overexpression of IRF7 was also shown to reduce DTMUV replication significantly, possibly through the activation of IFNα/β (Chen et al., 2019). As IRF3 is missing in avian species, IRF7 would play a major role in the induction of type I IFNs in virus-infected avian cells. Our transcriptomic data also showed that IFR7 was firstly induced by DTMUV infection, followed by a significant induction of IFNα/β in a later time point, supporting the regulatory role of IRF7-induced IFNα/β expression in DTMUV infection.

IFNs can activate the transcription factor ISGF3, which in turn binds to the ISRE of the IFIT5 gene promoter after nuclear entry, activating the transcription and translation of IFIT5. RNA and DNA viruses can be recognized by the corresponding PRRs, leading to the initiation of downstream signaling pathways and induction of IFIT5 in an interferon-dependent or non-interferon-dependent manner (Rauch et al., 2014). This is consistent with our observation that overexpression of IRF1 regulates IFIT5 expression.

VIPERIN and CMPK2 are co-transcribed by IFN stimulation. CMPK2 kinase belongs to the nucleoside monophosphate (NMP) kinase family and is a mitochondrial NMP kinase that can phosphorylate CMP and dCMP (Xu et al., 2008). The expression of CMPK2 is up-regulated during various viral infections (Zhang et al., 2014; Kommadath et al., 2017), implicating that VIPERIN and CMPK2 are functionally connected. VIPERIN may use different mechanisms to inhibit the replication of viruses (Seo et al., 2011; Teng et al., 2012; Fenwick et al., 2017). For example, VIPERIN inhibits the germination and release of influenza A virus and HIV-1 by binding to dialkyl telephosphate synthase to disrupt the lipid rafts (Seo et al., 2011). It can also interact with TBEV NS3 protein, resulting in the proteasome-dependent degradation of NS3, together with prM, E, NS2A, and NS2B (Panayiotou et al., 2018). In addition, VIPERIN can catalyze the conversion of cytidine triphosphate (CTP) to form structurally similar analogs 3′-deoxy-3′,4′-didehydro-CTP (ddhCTP), a substrate for most flavivirus RNA-dependent RNA polymerases (Gizzi et al., 2018). Incorporation of ddhCTP into RNA prevents further extension of the RNA template, thereby inhibiting virus replication (Minton, 2018). However, more recent studies showed that, rather than acting as a chain terminator, ddhCTP may restrict viral replication through inhibition of the NAD^+^-dependent enzymatic activities and regulation of inflammatory responses, including the cellular level of TNF-α (Van Gool et al., 2009; Honarmand et al., 2020). Inhibition of NAD^+^-dependent enzymatic reactions by ddhCTP may also induce the ADP ribosylation, promoting the degradation of viral proteins (Ullrich et al., 1999). Furthermore, ddhCTP may reduce the mitochondrial respiration and affect the nucleotide metabolism, resulting in reduced viral replication (Ebrahimi et al., 2020). Previous studies also suggest a correlation between VIPERIN and CMPK2 in the antiviral functions, and the main role of CMPK2 would be to ensure that CTP is not restricted in the viral replication site when VIPERIN is up-regulated (Minton, 2018). CMPK2 is a mitochondrial protein (Xu et al., 2008), while VIPERIN is cytosolic. Up-regulation and activation of CMPK2 may lead to depletion of nucleotides in the mitochondria and disturbance of mitochondrial homeostasis and functions, consequently influencing viral replication. Our results demonstrated that IRF1 induced VIPERIN transcription by directly binding to the VIPERIN promoter in DTMUV-infected cells. IRF1 may also participate in the regulation of IFIT5 expression, but not CMPK2. The induction kinetics presented in this study suggests that CMPK2 may be induced through the IFNα/β signaling pathway. In fact, it was reported that CMPK2 may function as an HIV restriction factor regulated by type I IFNs (El-Diwany et al., 2018).

VIPERIN can be induced in a variety of mammalian and avian cells, especially monocytes, upon stimulation with virus infection, IFN or other factors (Teng et al., 2012). In this study, we showed that huge different levels of VIPERIN expression were induced by DTMUV infection of different types of culture cells. For example, up to a 6000-fold induction of VIPERIN was observed in DTMUV-infected DF1 cells, but only 11-fold induction of the protein was detected in DTMUV-infected HEK293T cells. In addition to the differences in viral replication efficiency, certain regulatory pathways may be either absent or suppressed in some cell types. In fact, it was previously reported that VIPERIN could not be detected in HEK293T cells both in the presence and in the absence of IFNs (Gizzi et al., 2018). On the contrary, overexpression of a fusion construct containing the full-length MAVS and a truncated form of the Epstein-Barr virus protein LMP1 was shown to significantly induce the expression of VIPERIN in HEK293T cells (Gupta et al., 2016). Nevertheless, the weak induction of VIPERIN in DTMUV-infected HEK293T cells presented in this study would support the existence of a pathway(s) that leads to the induction of VIPERIN in this cell type. Most likely, IRF1 activated by DTMUV infection, as shown in this study, may play a direct role in up-regulation of VIPERIN in HEK293T cells.

In addition to infecting ducks, DTMUV can also infect geese (Ti et al., 2015; He et al., 2017), chickens (Fu et al., 2016), sparrows (Tang et al., 2013b), and mice (Li et al., 2013). Previous studies have shown that DTMUV not only can proliferate in poultry cells, but also exhibit cytopathic effects in various mammalian cells (Wang et al., 2016). In this study, we confirm the replication of DTMUV in BHK, 293T, Vero, DF1 and DEF cells, especially with high replication efficiencies in DEFs, DF1 and BHK cells.

In summary, this study has revealed, through genome-wide transcriptomic analysis and subsequent verification by RT-qPCR and Western blot analysis, the activation of a number of host antiviral innate immune signaling pathways in cells infected with DTMUV. Further functional characterization unravels an important function of IRF1 in the up-regulation of VIPERIN and restriction of DTMUV replication. Information presented in this study would guide future studies on DTMUV-host interactions, the molecular pathogenesis of DTMUV and cellular targets for developing novel antivirals.

## Data Availability Statement

The original contributions presented in the study are publicly available. This data can be found here: https://www.ncbi.nlm.nih.gov/sra/?term=SRP275736.

## Author Contributions

RC and DL designed and organized the study. CX did most of the experimental work. TX, LL, and FR did part of the experimental work. MH analyzed the data. CX, MH, and DL wrote the manuscript. All authors contributed to the article and approved the submitted version.

## Conflict of Interest

MH and RC were employed by the company Zhaoqing Institute of Biotechnology Co., Ltd. The remaining authors declare that the research was conducted in the absence of any commercial or financial relationships that could be construed as a potential conflict of interest.

## Footnotes

1 https://github.com/jstjohn/SeqPrep

2 https://github.com/najoshi/sickle

3 http://ccb.jhu.edu/software/tophat/index.shtml

4 http://deweylab.biostat.wisc.edu/rsem

5 http://www.bioconductor.org/packages/2.12/bioc/html/edgeR.html

6 https://github.com/tanghaibao/Goatools

7 www.e-crisp.org/E-CRISP

8 https://imagej.nih.gov/ij/

## Supplementary Material

FIGURE S1 Up-regulated genes in pathways activated in DTMUV-infected DEF cells.

FIGURE S2 Nucleotide sequence of the VIPERIN gene isolated from DF1-VIPERIN-KO cells. The DNA fragment was amplified from the genomic DNA extracted from DF1-VIPERIN-KO cells using primers (F:GTAACTGCCATGGTCATAATATCATAA; R:TGCACACTATTTTCTTACCTTCCA) and Taq polymerase. This fragment was then inserted into pMD19-T vector and sequenced with M13F and M13R primers. The top line represented the nucleotide sequence of wild type viperin. An extra adenosine (A) between A_950_ and C_951_ was inserted into the viperin gene in the knockout cell clone, resulting in frameshifting during translation. The sgRNA-targeted sequence was underlined.

FIGURE S3 Comparison of the growth kinetics and viability of DF1 and DF1-VIPERIN-KO cells. DF-1 and DF1-VIPERIN-KO cells were plated in 35 mm plates and measured at OD 450 absorbance after treating with CCK-8 (Thermo) at the indicated time points.

TABLE S1 The list of primer sequences.
