# Supplementary material for: Transcriptomic Analysis and Functional Characterization Reveal the Duck Interferon Regulatory Factor 1 as an Important Restriction Factor in the Replication of Tembusu Virus
Source: Front Microbiol. 2020 Aug 26;11:2069. doi: 10.3389/fmicb.2020.02069 (PMC7480082; doi:10.3389/fmicb.2020.02069)

Supplementary data

Table S1. The list of primer sequence

Primer name	Primer sequence (5′-3′)
Chicken GAPDH-F	GCCATCACAGCCACACAGA
Chicken GAPDH-R	TTTCCCCACAG CCTTAGCA
Chicken VIPERIN-F	TCGTTCTGCCTCTGCTCTCCTG
Chicken VIPERIN-R	TTGTAGTTGCACTGCCTGGT GAAG
Chicken IFIT5-F	CACCAGCTAGGACTCTGCTACCG
Chicken IFIT5-R	CCTCCGCATACATC CTTGCCAAG
Chicken CMPK2-F	ATCGGTGCTGGACATCCTGGAG
Chicken CMPK2-R	GCAAGCTGG CGGAGACCTTAAC
Chicken IRF1-F	AAGGAGCAGGACGGCGAGATC
Chicken IRF1-R	ACGGTGT CCAGCCAGGAGAAG
Human GAPDH-F	CTGGGCTACACTGAGCACC
Human GAPDH-R	AAGTGGTCGTTGAG GGCAATG
Human VIPERIN-F	TTGCATTGCTTTGTTCGCCT
Human VIPERIN-R	TATGCCAA CCCAGTGTAACG
Human IFIT5-F	ATGGCCGCTTTCAGGAATTTCACC
Human IFIT5-R	AGCACTTG TCAGTTTGGTGCGAAG
Human CMPK2-F	AATTTGGGACTGAGGGAGATG
Human CMPK2-R	CTAC ACTGGCATGCTGATGA
Duck GAPDH-F	ATGAGAAGTATGACAAGTCC
Duck GAPDH-R	ACTGTCTTCGTGTG TGGCT
Duck RIG-I-F	GCTACCGCCGCTACATCGAG
Duck RIG-I-R	TGCCAGTCCTG TGTAAC CTG
Duck MDA5-F	GCTACAGAAGATAGAAGTGTCA
Duck MDA5-R	CAGGATCAGA TCTGGTTCAG
Duck TLR3-F	GAGTTTCACACAGGATGTTTAC
Duck TLR3-R	GTGAGATTTGT TCCTTGCAG
Duck TLR7-F	GTTCAAGCGATTCCAAGCTC
Duck TLR7-R	GCAACTTCGGCCA TACTCAT
Duck IFN-α-F	TCCTCCAACACCTCTTCGAC
Duck IFN-α-R	GGGCTGTAGGTGTGG TTCTG
Duck IFN-β-F	AGATGGCTCCCAGCTCTACA
Duck IFN-β-R	AGTGGTTGAGCTGGTTG AGG
Duck MX-F	GCTGTCCTTCATGACTTCG
Duck MX-R	GCTTTGCTGAGCCGATTAAC
Duck PKR-F	AATTCCTTGCCTTTTCATTCAA
Duck PKR-R	TTTGTTTTGTGCCATATCTTG
Duck MHC-I-F	GAAGGAAGAGACTTCATTGCCTT
Duck MHC-I-R	TCTCCTCTCCAGTACGTC CTTCC
Duck MHC-II-F	CACCTTTACCAGCTTCGAG
Duck MHC-II-R	CCGTTCTTCATCCAGGT GA
Duck IL-1β-F	TCATCTTCTACCGCCTGGACG
Duck IL-1β-R	TAGGTGGCGATGTTGAC CT
Duck IL-2-F	CCAAGAGCTGACCAACTTC
Duck IL-2-R	ATCGCCCACACTAAGAGCA
Duck IL-6-F	TTCGACGAGGAGAAATGCTT
Duck IL-6-R	CCTTATCGTCGTTGCCAGAT
Duck IL-8-F	AAGTTCATCCACCCTAAATC
Duck IL-8-R	GCATCAGAATTGAGCTGAG
Duck VIPERIN-F	GCCGAGAGTATGCTGTTGCTT
Duck VIPERIN-R	AATGAGCAGGCACTGGAACAC
Duck IFIT5-F	AAGCTACCTTCAAACGGGTA
Duck IFIT5-R	TCCTCCTTCAGCAAAGTCCA
Duck CMPK2-F	CCTGGATGCCGTTCTGTTAAGGTC
Duck CMPK2-R	GCTGTGCTGTGCCAGTATCTGTC
Duck IRF1-F	GAACTCCCTGCCTGACATCG
Duck IRF1-R	CTCTTGCTCCTGCTTCTTGCTT
DTMUV-e-F	CGCTGAGATGGAGGATTATGG
DTMUV-e-R	ACTGATTTTTGGTGG CGTG

Figure S1. The up-regulated genes in pathways activated in DTMUV infected DEF cells.

A. Toll- like receptor signaling pathway

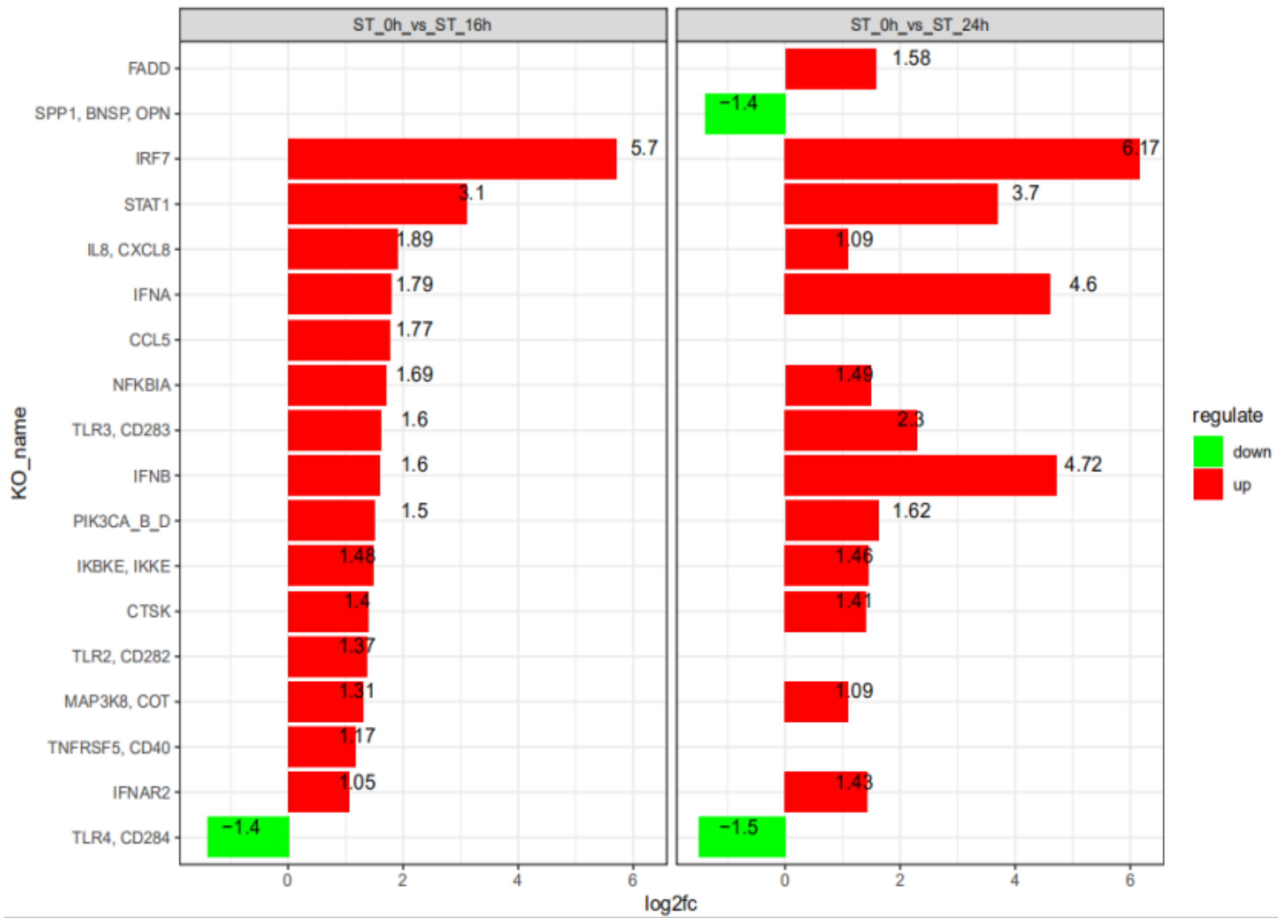

Figure S1. The up-regulated genes in pathways activated in DTMUV infected DEF cells.

B. Rig-I-like receptor signaling pathway

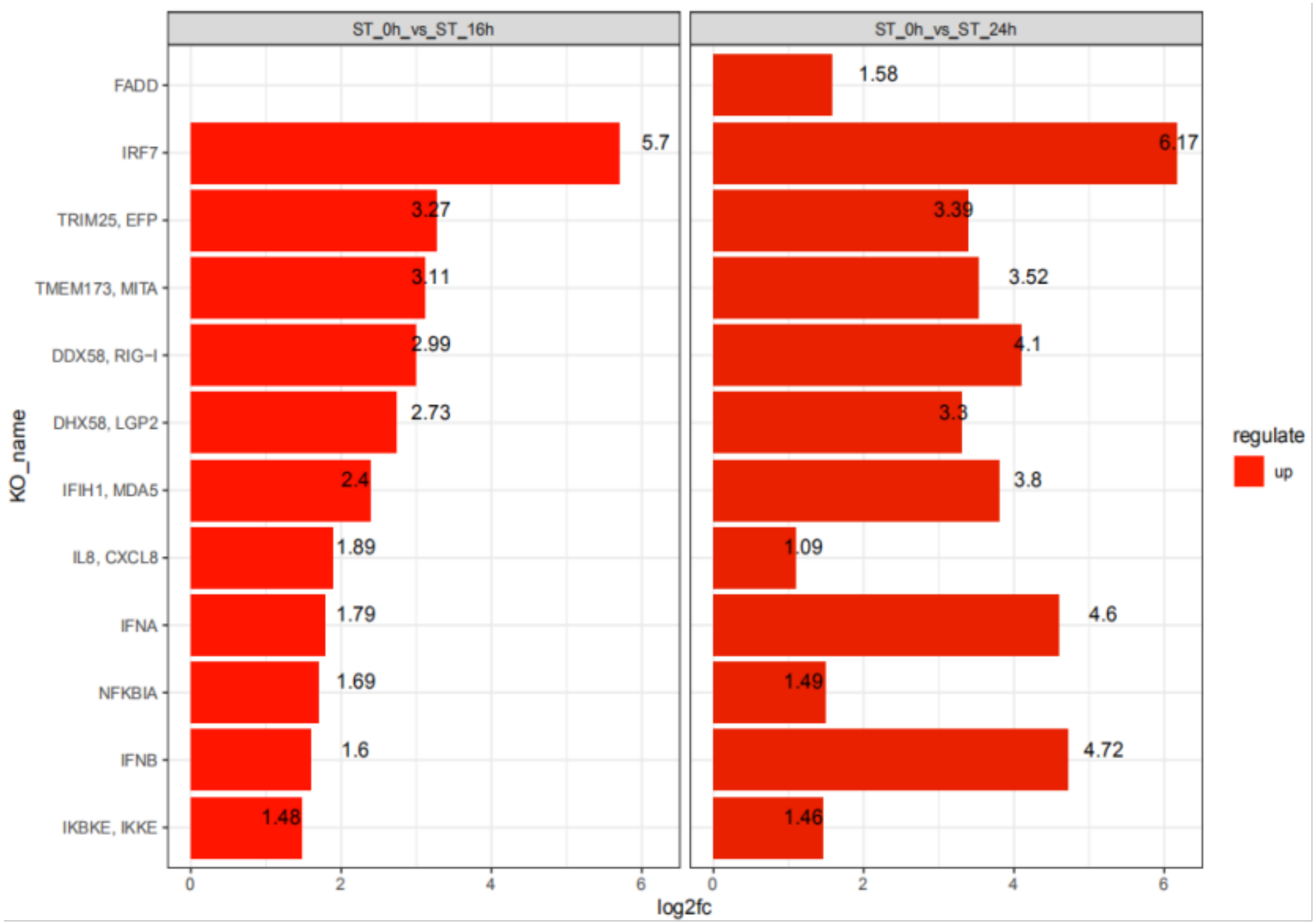

Figure S1. The up-regulated genes in pathways activated in DTMUV infected DEF cells.

C. Nod-like receptor signaling pathway

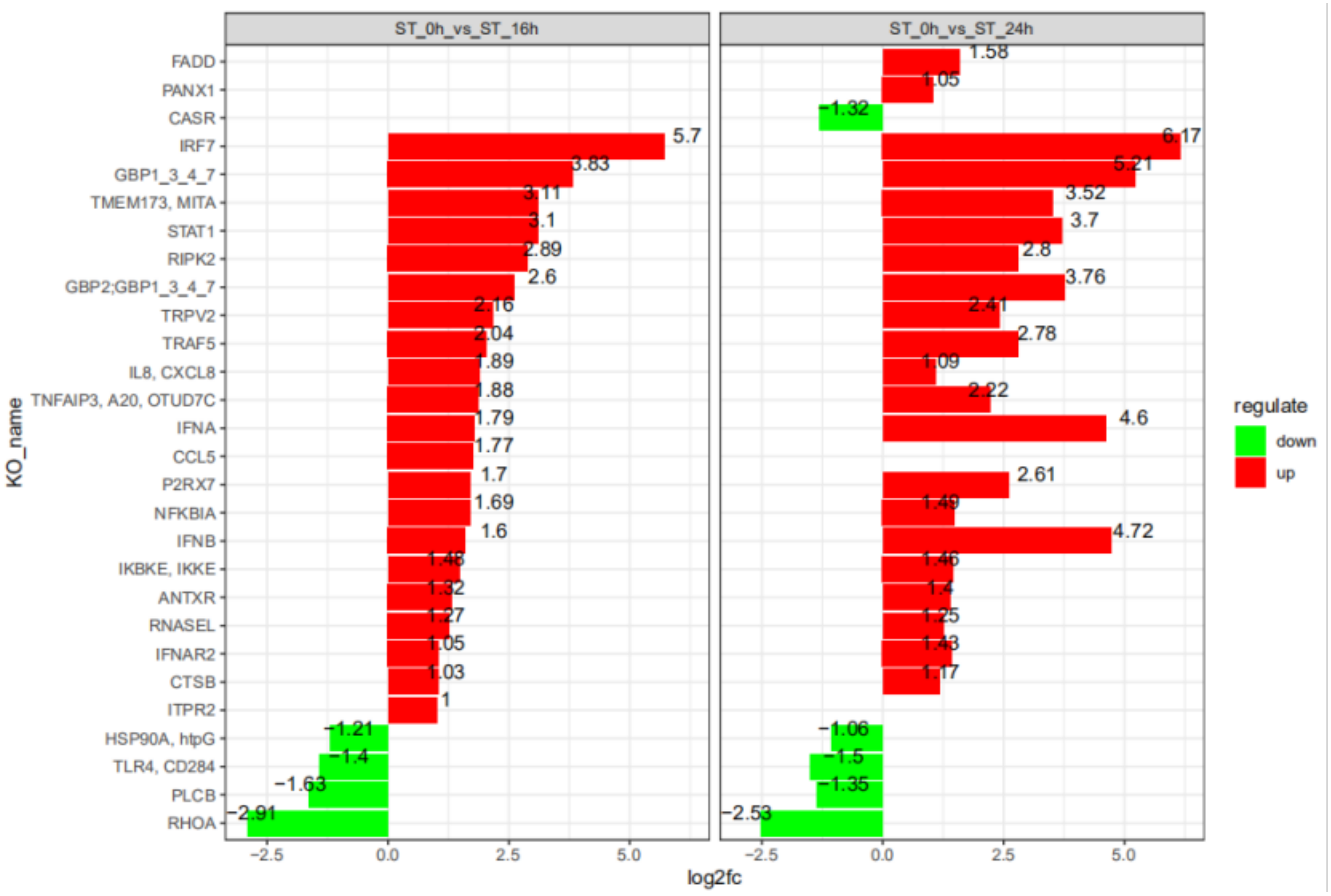

Figure S1. The up-regulated genes in pathways activated in DTMUV infected DEF cells.

D. Chemokine signaling

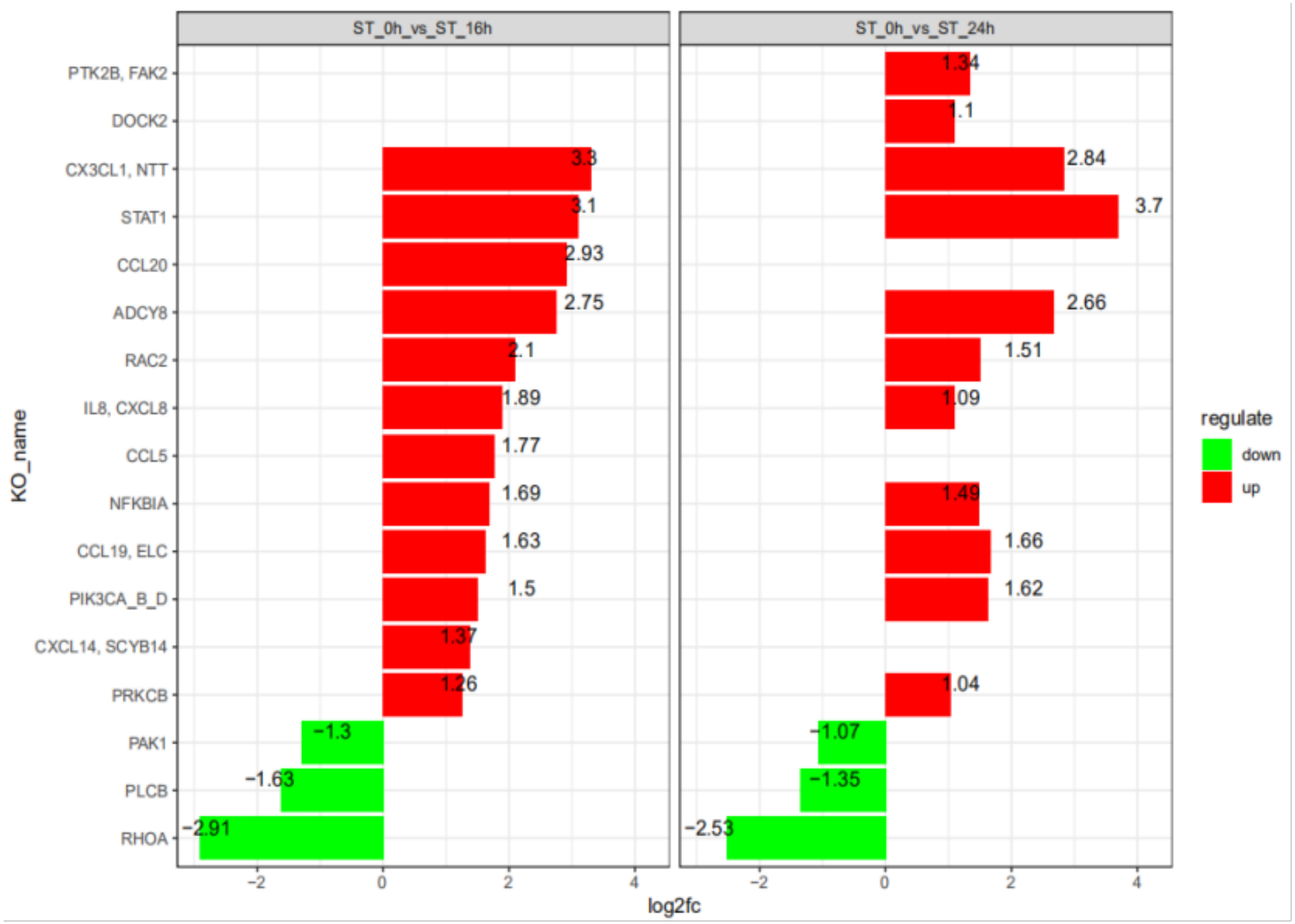

Figure S1. The up-regulated genes in pathways activated in DTMUV infected DEF cells.

E. Hepatitis signaling

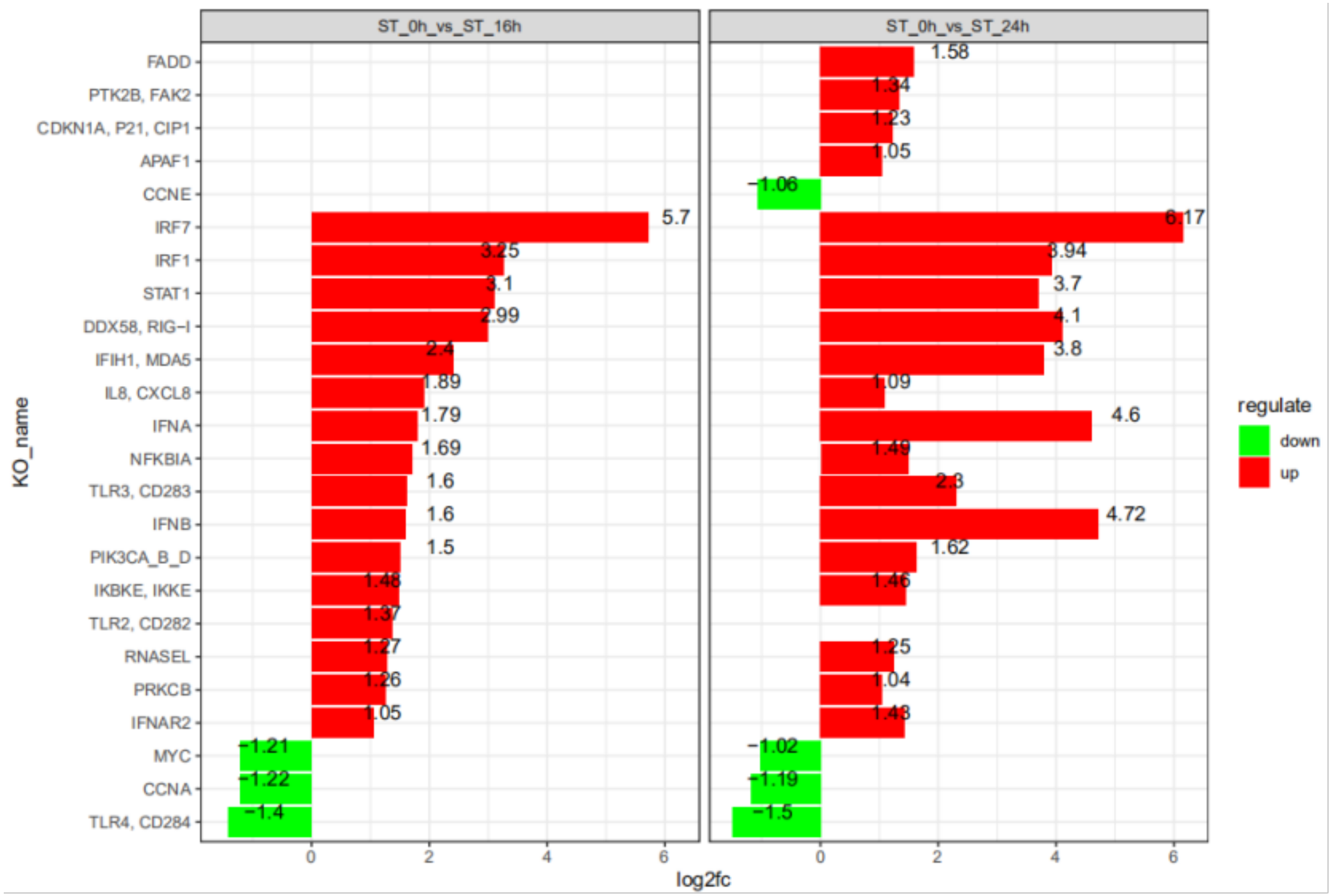

Figure S2. The sequence of the VIPERIN gene isolated from DF1-VIPERIN-KO cells

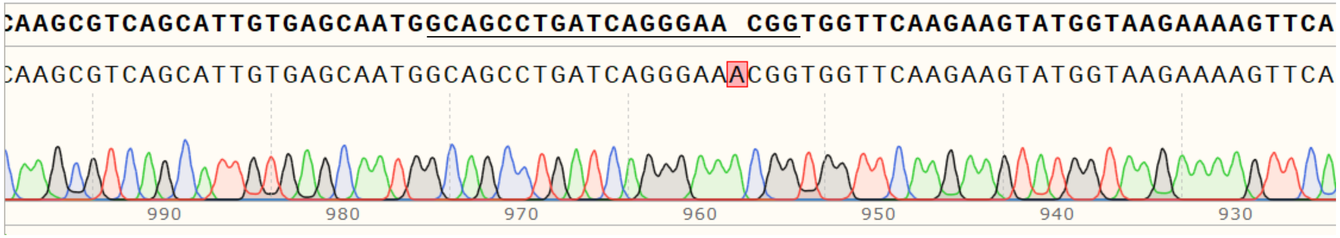

Figure S3. Comparison of the growth kinetics and viability of DF1 and VIPERIN-knockout-DF1 cells

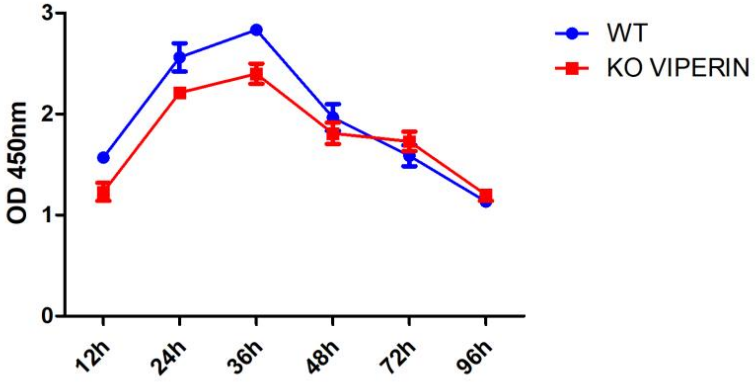

Supplement: FIGURE S1 — Up-regulated genes in pathways activated in DTMUV-infected DEF cells. [file Data_Sheet_1.PDF]
